# Motivations, sources of influence and barriers to being a podiatrist: a national questionnaire of student views

**DOI:** 10.1186/s13047-022-00551-6

**Published:** 2022-05-28

**Authors:** Lucy Wallis, James Faulkner, Rachel Locke, Beverley Harden, Emma E. Cowley

**Affiliations:** 1 University of Winchester, Sparkford Road, Winchester, SO22 4NR UK; 2 Health Education England, Southern House, Otterbourne, Winchester, SO21 2RU UK; 3 University of Southampton, University Road, Southampton, SO17 1BJ UK

**Keywords:** Podiatry, Students, Career choice, Sources of influence, Barriers, Questionnaire, Mature students

## Abstract

**Background:**

Podiatry is an allied health profession which has seen a substantial decline in numbers in recent years. Every effort is required to recruit more students to reverse this diminishing supply and meet national foot health needs. To increase the number of applications to podiatry courses and encourage individuals to choose podiatry careers, the aim of this study was to understand the key motivations, sources of influence and barriers to choosing a podiatry career among current podiatry students, and consider the influence of choosing podiatry before or after a first career.

**Methods:**

An online questionnaire, comprising mainly Likert-scale questions, was disseminated to podiatry students in England between February and March 2021. Respondents to the questionnaire were categorised as individuals who had either decided to engage in the profession ‘before’ or ‘after’ a first career. Mann-Whitney U non-parametric difference tests were performed to compare outcome questions relating to motivations, sources of influence and barriers between groups.

**Results:**

One hundred and fifteen students completed the questionnaire. Overall, the study demonstrated many similarities between the groups (before and after a first career). However, there were distinct differences when considering some of the motivations (i.e., intellectually stimulating, student bursaries), sources of influence (i.e., own patient experience) and barriers (i.e., financial, job availability) associated with engaging in the podiatry profession. Overall, altruistic reasons were the key motivations for choosing podiatry. Personal sources of influence such as conducting own research, was the most important source of influence. Similar to other studies, a lack of awareness of the podiatry profession and what it entails remains problematic.

**Conclusions:**

This is the first national questionnaire investigating career choice decision-making for podiatry students in England or in any other country. The similarities suggest that marketing is applicable to both groups. However, an absolute must is a future national strategy that makes educational sources more impactful. Additionally, following the Covid-19 pandemic, the increased interest in health and care professions suggests now is the right time to market podiatry to individuals looking for a career change. Finally, the influence of personal encounters with podiatrists shows the transformational role podiatrists can have in recruiting to the profession.

**Supplementary Information:**

The online version contains supplementary material available at 10.1186/s13047-022-00551-6.

## Background

With an ageing population with growing health and social care needs [1], there is an ever increasing demand on the National Health Services (NHS) podiatry departments [2, 3]. However, recruitment of podiatrists falls short of NHS workforce requirements [4]. With the predicted supply of podiatrists in England being insufficient to meet foot health needs in the next ten years, ‘every effort’ is required to recruit more students to reverse the diminishing supply of podiatrists [4]. Applications and acceptances to podiatry pre-registration courses have been in decline since 2012. This was exacerbated in 2016 with the removal of the non-repayable NHS bursary which covered podiatry students’ university fees and which was replaced with tuition fees and loans in 2017, and the addition of the Learning Support Fund in 2020 [5]. The cessation of national commissioning of healthcare training led to an increase in healthcare programmes e.g. physiotherapy which have attracted many students who may previously have considered podiatry. Undergraduate podiatry entrants decreased by 19% in 2017–18 in comparison to 2016–17 [6] and there was a 40% reduction overall between 2017 and 2019 [4]. Furthermore, there was an 8.1% attrition rate from the Health and Care Professions Council (HCPC) podiatry register [7]. The Saks report highlighted that there had been an increase in podiatry student recruitment [4] but it is important to acknowledge the effect of the Covid-19 pandemic on fluctuations in podiatry course entrants from the past two years.

Addressing the reduction in the podiatry workforce, alongside other allied health professionals (AHPs), was recognised in the NHS Long-Term plan as part of the new workforce implementation plan [8]. If universities continue to experience issues with student recruitment to AHP courses, they may become unsustainable leading to a vacuum of qualified professionals which will affect delivery of care [9] and may lead to failure of the profession. It should be acknowledged that podiatry is one of four AHPs, alongside therapeutic radiography, prosthetics and orthotics and orthoptics, seen as being vulnerable to university course recruitment [9]. The landscape of AHP numbers and recruitment is varied and there are professions with more success in recruiting students to their profession than others.

In a recent study, Whitham et al., [10] generated four themes from focus group discussions with Generation Z participants about what attracted them to podiatry careers. These were lack of awareness of podiatry, accessibility of course and career, career status and breadth/opportunity of scope of practice. To increase the applications to podiatry courses, it is important to understand the decision-making process in choosing this career. A podiatry career offers a variety of practice settings including working in the NHS, the private sector or freelance, while pre-registration training includes general medicine, pharmacology, infection control and public health [4]. A strong characteristic of podiatry student cohorts is diversity including age. In comparison to other AHP courses, there is a higher proportion of mature students (~ 48 years) choosing podiatry courses and/or individuals who have enrolled in podiatry courses following a professional career in a different area of expertise [11]. Accordingly, in 2016/2017, 45% of students beginning a podiatry undergraduate course in England were over 25 years of age [6].

Using a national questionnaire administered across England, the purpose of this study was to: i) identify key motivations, sources of influence and barriers to choosing a podiatry career among current podiatry students in England, and ii) consider the influence of choosing podiatry as a first career or after a first career, on motivations, sources of influence and barriers. This is the first study to explore these topics and draws on the results of this national questionnaire to understand students’ choice to become a podiatrist and to identify practical implications for educators and those responsible for future workforce strategy and transformation. It seeks to meet a key imperative to increase recruitment to the podiatry profession.

## Methods

This study reports the national results in the area of podiatry from an online questionnaire designed and hosted using JISC (Bristol, UK) software which was disseminated to AHP students for four weeks between February and March 2021. The convenience sample for this study was students currently enrolled on all undergraduate and postgraduate podiatry courses in England. Gatekeepers were in the form of Education Leads for the professional body who distributed the questionnaire to universities in England. Participants were sent a link to access the questionnaire. Additionally, the questionnaire was promoted through the Health Education England (HEE) website, blog posts on HEE connect, HEE internal newsletters, a webpage detailing the project [12], social media and newsletters of the professional body.

Ethical approval for the study was obtained from the University of Winchester Research and Knowledge Exchange Ethics Committee (Reference: HWB_REC_21_03). A participant information page explaining the study was included at the beginning of the questionnaire. Participants then confirmed consent through ticking a confirmation box. The questionnaire was anonymous and took approximately fifteen minutes to complete.

The questionnaire was designed based on the findings of a scoping review [13] and focus groups conducted with members of an AHP leadership programme. This process also informed the content validity of the questionnaire. The questionnaire was piloted among physiotherapy students at the University of Winchester and members of the AHP leadership programme. In total, 40 AHP students piloted the questionnaire. The piloting process helped establish face validity and ensured the questionnaire was relevant for all AHPs.

Participants were asked about their background, and the motivations, sources of influence and barriers to choosing an AHP career. A series of questions were posed within these broad headings, with participants being provided the opportunity to respond on a Likert scale. The Likert scale included the following statements and numeric values: strongly disagree (1), disagree (2), neutral (3), agree (4), strongly agree (5). Participants could also respond to any given question with a not-applicable response, while there was also the opportunity to add additional context to the answers provided in the questions via a series of free-text boxes. Open-ended questions included asking about public perception of their profession and advice to individuals interested in the profession. Demographic questions included year of study, ethnicity, disability, gender and age. An additional file shows the questionnaire (see Additional file 1).

### Data analysis

As a result of the relatively high levels of podiatry students over the age of 25 [6], respondents were categorised as individuals who had either engaged in the profession ‘before’ or ‘after’ their first career in employment (Pre-FC and Post-FC, respectively). Pre-FC respondents included individuals who made their decision to join the podiatry profession during their secondary school, college or during their initial University degree. Post-FC respondents included individuals who were previously employed in an alternative career before joining the podiatry profession. Prior to statistical analysis, N/A responses on Likert scales were removed from the analysis. NVivo was used for managing the open-ended question data. The data was initially filtered for Pre-FC and Post-FC. Thematic analysis using Braun and Clarke’s approach [14] was utilised to analyse the open-ended questions. This involved becoming familiar with the data through reading and re-reading the open-ended question responses. The next step was generating initial codes. This took place through Inductive coding and themes were then identified from groups of codes. The final step in the process was defining and naming the emergent themes. The main themes were an interpretation of the open-ended question data obtained, which had allowed for participants to share their perspectives using their own words.

### Statistical analysis

Demographic and outcome data was checked for normality using tests for skewness and kurtosis, as well as a graphical assessment for normal distribution. Thereafter, Mann-Whitney U non-parametric difference tests were used to compare participants’ age, gender, ethnicity, year of study and disability between Pre-FC and Post-FC groups. A series of Mann-Whitney U tests were also used to compare outcome questions relating to personal, professional interests and day-to-day job context motivations; personal and educational, media and marketing sources of influence; and personal, professional and understanding the role barriers between groups. Data is presented as median and interquartile range (IQR; 25th–75th percentiles), mean ranks for Pre-FC and Post FC, U statistic, z score and *p* value. Effect sizes are also reported as r based on the following formula:

$$r=\frac{z}{\surd n}$$

Whereby z is the z score and n is the number of participants. Effect sizes of 0.1, 0.3 and 0.5 denote a small, medium and large effect, respectively. Statistical significance was originally set at *p* < 0.05 but adjusted where necessary via the Bonferroni technique to minimise the risk of type I error. Statistical analysis was undertaken on SPSS (v.26).

## Results

This questionnaire recruited 115 podiatry participants (Pre-FC, *n* = 50; Post-FC, *n* = 65; Table 1). This is a response rate of 12.8% among a population of approximately 900 students. Pre-FC participants were typically younger and of a more varied ethnicity compared to Post-FC participants (both *p* < 0.01; Table 1). Post-FC participants were generally older (36+ years) and of white ethnicity (85%). There were no differences in gender, type of study, year of study and number of respondents with some form of disability between Pre-FC and Post-FC respondents (*p* > 0.05; Table 1). The results begin with the findings from the demographic and Likert scale questions before focusing on the open question findings.

**Table 1:** Participant demographics of Pre-FC and Post-FC participants. Data is presented as n and percentage of sample

	Pre-FC	Post-FC	***P***
**Participants (n)**	50	65	
**Age range (y)**			
< 21	20 (40%)	0 (0%)	**.000**
21–25	17 (34%)	4 (6%)	
26–30	4 (8%)	9 (14%)	
31–35	4 (8%)	6 (9%)	
36–40	4 (8%)	14 (22%)	
41–45	1 (2%)	16 (25%)	
46–50	0 (0%)	8 (12%)	
Over 51	0 (0%)	8 (12%)	
**Gender**			
Male	7 (14%)	8 (12%)	.790
Female	43 (86%)	57 (86%)	
**Ethnicity**			
White	31 (62%)	55 (85%)	**.009**
Mixed or multiple ethnic background	1 (2%)	2 (3%)	
Asian	11 (22%)	4 (6%)	
Black	5 (10%)	4 (6%)	
Arab	2 (4%)	0 (0%)	
**Study**			
Full-time	49 (98%)	62 (95%)	.450
Part-time	1 (2%)	3 (5%)	
**Disability**			
Yes	11 (22%)	12 (18%)	.706
No	39 (78%)	53 (82%)	

*Note*: *Pre-FC* before first career, *Post-FC* after first career

Bold *p* values denotes significant difference between groups (*p* < 0.05)

### Motivations, sources of influence and barriers to choosing podiatry (Aim 1)

Participants identified *someone in the profession I saw/met who was a really good role model for me* and *my own research* into the podiatry profession as two important personal sources of influence (Table 2). In terms of motivations for choosing podiatry, particularly with regards to the day-to-day context of the profession, participants typically agreed/strongly agreed with all of the items reported (see Additional file 2). *Where I can use my skills to improve the quality of life for a patient/service user* was the most important motivation for both groups (see Additional file 2). *Academic interests*, *interest in area of profession*, *intellectually stimulating* and *that suits my personal qualities and values* were all perceived to be important motivations for engaging in the profession (Table 3). With regards to professional motivations, excluding the *student bursary item*, participants agreed/strongly agreed with all items (e.g., the *potential for job security*, *the opportunity for working in the private or public sector*, *good job availability and employment opportunities*; Table 4). With regards to barriers to the profession, lower scores were generally reported when compared to the aforementioned areas of interest, with the median score for all items being ≤4 (see Additional file 2).

**Table 2:** Personal and educational sources of influence of Pre-FC and Post-FC participants

	Group	Median (IQR)	Mean rank	U	Z	***p***	Effect size (r)
**Previous healthcare job**	**Pre-FC**	3 (2–4)	33.00	500	−1.060	0.289	0.12
	**Post-FC**		38.36				
**Role model**	**Pre-FC**	4 (3.75–5)	51.00	1254	−0.158	0.875	0.02
	**Post-FC**		51.88				
**Family member**	**Pre-FC**	4 (2–5)	41.79	738	−0.442	0.658	0.05
	**Post-FC**		39.54				
**Friend**	**Pre-FC**	3.5 (2–4)	34.88	588	−1.544	0.123	0.17
	**Post-FC**		42.72				
**Works with profession**	**Pre-FC**	4 (2–4.75)	42.04	856.5	−0.162	0.871	0.02
	**Post-FC**		42.88				
**Own patient experience**	**Pre-FC**	4 (2–5)	38.64	727	−1.996	0.046*	0.21
	**Post-FC**		49.16				
**Own research**	**Pre-FC**	4 (4–5)	54.13	1248.5	−0.520	0.603	0.05
	**Post-FC**		51.31				
**Teacher**	**Pre-FC**	2 (2–4)	38.57	400	−2.078	0.038*	0.25
	**Post-FC**		29.00				
**Professional visiting school/college**	**Pre-FC**	2 (1–3)	38.46	341.5	−2.549	0.011*	0.32
	**Post-FC**		27.02				
**Future careers programme**	**Pre-FC**	2 (1–3)	35.02	314.5	−2.102	0.036*	0.27
	**Post-FC**		25.98				
**Careers advise professional**	**Pre-FC**	2 (1–3)	34.44	389.5	−1.356	0.175	0.17
	**Post-FC**		28.56				
**Voluntary work**	**Pre-FC**	2 (2–4)	39.50	608	−0.847	0.397	0.10
	**Post-FC**		35.39				
**Shadowing experience**	**Pre-FC**	4 (2–5)	41.04	815.5	−0.165	0.869	0.02
	**Post-FC**		41.88				
**First University degree**	**Pre-FC**	2 (2–40	36.86	478.5	−0.775	0.438	0.09
	**Post-FC**		33.13				

*Note*: *IQR* interquartile range (25th – 75th percentiles), *Pre-FC* before first career, *Post-FC* after first career

*Denotes *p* values that are approaching a significant difference between groups following a Bonferroni adjustment (*p* = .003)

**Table 3:** Personal motivations for engaging in podiatry for both Pre-FC and Post-FC participants

	Group	Median (IQR)	Mean rank	U	z	***p***	Effect size (r)
**Reflects academic interests**	**Pre-FC**	4 (4–5)	55.44	1497.0	−0.639	0.523	0.06
	**Post-FC**		59.11				
**Interest in area of profession**	**Pre-FC**	4 (4–5)	52.3	1340.0	−1.621	0.105	0.15
	**Post-FC**		61.56				
**Challenging role**	**Pre-FC**	4 (3–5)	51.43	1296.5	−1.983	0.047*	0.18
	**Post-FC**		63.05				
**Intellectually stimulating**	**Pre-FC**	4 (4–5)	50.1	1230.0	−2.332	0.020*	0.22
	**Post-FC**		63.28				
**High profile responsibilities**	**Pre-FC**	4 (3–5)	54.79	1464.5	−0.943	0.346	0.09
	**Post-FC**		60.47				
**Personal qualities and values**	**Pre-FC**	5 (4–5)	50.73	1259.0	−1.555	0.120	0.15
	**Post-FC**		59.19				
**Good public image**	**Pre-FC**	4 (3–4)	53.3	1382.5	−0.935	0.350	0.09
	**Post-FC**		58.9				
**Respected in culture**	**Pre-FC**	3 (3–4)	45.71	1019.5	−1.220	0.223	0.12
	**Post-FC**		52.46				
**Suits religious background**	**Pre-FC**	3 (2–3)	40.57	609.5	−1.031	0.302	0.12
	**Post-FC**		35.63				
**Supportive attitude**	**Pre-FC**	3 (3–4)	40.58	801.0	−1.001	0.317	0.11
	**Post-FC**		45.81				

*Note*: *IQR* interquartile range (25th – 75th percentiles), *Pre-FC* before first career, *Post-FC* after first career

*Denotes *p* values that are approaching a significant difference between groups following a Bonferroni adjustment (*p* = .005)

**Table 4:** Professional motivations for engaging in podiatry for both Pre-FC and Post-FC participants

	Group	Median (IQR)	Mean rank	U	z	***p***	Effect size (r)
**Vocational degree**	**Pre-FC**	4 (4–5)	54.20	1431	−0.847	0.397	0.08
	**Post-FC**		59.14				
**Student bursaries**	**Pre-FC**	3 (2–4)	47.26	1090.5	−2.490	0.013*	0.24
	**Post-FC**		62.12				
**Regular hours**	**Pre-FC**	5 (4–5)	53.34	1392	−1.495	0.135	0.14
	**Post-FC**		61.58				
**Salary**	**Pre-FC**	4 (3–4)	55.87	1512.5	−0.488	0.625	0.05
	**Post-FC**		58.73				
**Job security**	**Pre-FC**	5 (4–5)	52.67	1356	−1.510	0.131	0.14
	**Post-FC**		61.14				
**Entrepreneurial**	**Pre-FC**	4 (3–5)	58.1	1514	−0.325	0.745	0.03
	**Post-FC**		56.16				
**Employment opportunities**	**Pre-FC**	5 (4–5)	59.62	1439.5	−0.848	0.397	0.08
	**Post-FC**		54.99				
**Private sector**	**Pre-FC**	4 (4–5)	61.14	1414	−1.097	0.273	0.10
	**Post-FC**		54.75				
**Public sector**	**Pre-FC**	4 (4–5)	57.59	1539	−0.182	0.855	0.02
	**Post-FC**		56.55				
**Work in the NHS**	**Pre-FC**	5 (4–5)	60.43	1453.5	−0.925	0.355	0.09
	**Post-FC**		55.21				
**Work in healthcare**	**Pre-FC**	5 (4–5)	61.28	1461	−1.087	0.277	0.10
	**Post-FC**		55.48				
**Career advancement**	**Pre-FC**	5 (4–5)	57.02	1567	−0.006	0.995	0.00
	**Post-FC**		56.98				
**Further education**	**Pre-FC**	4 (4–5)	59.18	1466	−0.676	0.499	0.06
	**Post-FC**		55.27				
**Different work environments**	**Pre-FC**	5 (4–5)	55.7	1510	−0.566	0.571	0.05
	**Post-FC**		58.91				

*Note*: *IQR* interquartile range (25th – 75th percentiles), *Pre-FC* before first career, *Post-FC* after first career

*Denotes *p* values that are approaching a significant difference between groups following a Bonferroni adjustment (*p* = .004)

### Pre-FC and Post-FC effect on motivations, sources of influence and barriers to choosing podiatry (Aim 2)

#### Motivations

*Intellectually-stimulating* and *challenging role* were approaching statistical significance, with Post-FC participants reporting them to be more important to them than Pre-FC (Table 3). There were no other differences between groups when examining personal interests (all *p* > 0.05; Table 3). S*tudent bursaries* were also approaching statistical significance with the Post-FC group perceiving it to be more important than the Pre-FC group (Table 4). There were no differences in the perceived importance of the day-to-day context of the profession between groups (all *p* > 0.05; see Additional file 2).

#### Sources of influence

*Teacher, professional visits to school/colleges, careers programme run by school/colleges* and *careers fairs* were all more important for Pre-FC participants compared to the Post-FC participants (Table 2). However, Post-FC recognised *own patient experiences (their own experience or that of a relative receiving care from the profession)* as a more important personal influence than Pre-FC, with findings approaching statistical significance (Table 2). There were no other differences between groups in how information on podiatry was accessed (see Additional file 2).

#### Barriers

With regards to professional barriers, *job availability* was identified as a key barrier for the Post-FC group *(p < 0.05). Financial support* was approaching statistical significance, with a higher median rank reported for Post-FC participants (*p* = 0.08; see Additional file 2). There were no differences in any of the personal barriers between groups (all *p* > 0.05; see Additional file 2), although outside obligations was approaching statistical significance (*p* = 0.09). When trying to understand the profession, *careers advisors' lack of awareness of the profession* and *misconception of profession* were reported to be more important barriers for the Pre-FC group, with each of these analyses approaching statistical significance (*p* < 0.05; see Additional file 2).

#### Open questions responses

Two open-ended questions were included in the questionnaire. Table 5 illustrates a number of responses from the two groups. These have been selected as they represent the views of several participants in each group. In terms of public perception of podiatry, which was answered by 45% of the sample, themes identified included a lack of awareness and understanding about the profession and what the job entails which were highlighted by both groups. Any knowledge, and positive perception, was likely to be obtained through personal experience with a podiatrist. The perception of podiatry as ‘nail cutting’ was raised by a quarter of question respondents of the Post-FC group and it was suggested that this coincided with a view of podiatry not being taken seriously as a healthcare course. The second open-ended question asked what advice participants would give someone interested in the profession. This was answered by 81% of the sample. In terms of pre-application advice, a key theme was the importance of undertaking work experience or shadowing to understand the role which both groups emphasised. More participants in the Post-FC group mentioned being mindful of the academic workload and the financial commitment of the course.

**Table 5:** Showing Pre-FC and Post-FC responses to the two open questions

Question	Pre-FC group	Post-FC group
What do you feel is the public understanding of your profession (positive or negative aspects)?	*Most members of the public do not understand what we do and who we are.* *The general public seem to think it’s just nail cutting and callus removal, only patients directly involved in the services understand the role we play in healthcare. This view is also sadly shared with students from other professions that I have encountered so far during my studies.* *Podiatry- it’s just cutting toenails. It’s a bit more than that.* *I feel that those who have not used a podiatry service would not understand the profession and why we chose it.* *I feel a lot more education is needed as if people have an issue with their mouth, they head straight to the dentist, but if they have foot pain, they always go to the doctor, never straight to a podiatrist.*	*I don’t think people understand the full scope of a career in podiatry. Most people think of it as just toenail cutting but don’t realise that the career options within podiatry are so diverse.* *Not really aware of it until they are older or have a problem. Most people say ‘oh my god why do you want to touch feet’?.* *I think the majority of people are unaware of the extent of the training, knowledge and expertise of a podiatrist.* *For those who use the service very positive but for those who don’t mostly negative.* *The general public may not have a good understanding of podiatry profession as it is confused with the work of beauty salons or other foot practitioners with no degree.*
From what you know now as a student on your course, what advice would you give someone interested in this profession?	*Definitely to look into this profession … it is perfect for an academic student who may be a little burnt out from exams and looking for a blend of hands on and book- based assessments.* *To go for it as there are so many different paths to go down and progression is excellent. No day is the same and the range and variety of locations and treatments is fantastic.* *Get as much work experience as you can from private practice and NHS public settings to get a flavour of what it’s like working as a podiatrist.*	*Go and get as much information about the role as they can, I would also let them know about the heavy academic workload.* *See if you can organise work experience and ask questions.* *To definitely go for it. Be realistic about where you do your programme if you have other commitments that mean you can’t relocate to where your uni. is. It can get very expensive otherwise.* *Research funding opportunities available to undertake the course.*

## Discussion

The purpose of this study was to identify the key motivations, sources of influence and barriers to choosing a podiatry career for students, and whether this differed between people who had (not) previously engaged in an alternative first career (Pre-FC and Post-FC). Altruistic reasons were the key motivations for choosing podiatry. Personal sources of influence, such as seeing a podiatrist at work, *someone in the profession I saw/met who was a really good role model for me* or *my own research*, were the most important sources of influence. Overall, educational, media and marketing sources scored low in terms of influence. On the whole, potential barriers to the profession scored low perhaps owing to the fact that the participants had overcome these barriers to enable their engagement with the podiatry profession. Nevertheless, a lack of awareness of the podiatry profession and what it entails remains problematic. Although the study demonstrated many similarities between Pre-FC and Post-FC respondents across the main themes to the study, there were distinct differences between groups when considering some of the motivations (i.e., *intellectually stimulating*, *student bursaries*), sources of influence (i.e., *own patient experience*) and barriers (i.e., financial, *job availability*) associated with engaging in the podiatry profession. However, as only small to medium effect sizes were observed between groups, these findings must be interpreted with caution. As this is the first national questionnaire to explore these topics in England, these findings may have important implications for recruitment of podiatrists both at the national level, (e.g. NHS and HEE), but also at a local level for universities advertising podiatry courses, and school and colleges providing satisfactory information on podiatry for it to be seen as a viable career option for both Pre-FC and Post-FC students.

### Motivations

Post-FC participants reported *intellectually stimulating, challenging role* and *student bursaries* as three motivations that were of greater importance to them compared to Pre-FC. The two former motivations are likely related to Post-FC participants wanting to commit to a career change that may ultimately lead to job satisfaction [15]. In promoting podiatry, the importance of these motivations suggests that there needs to be a greater focus on the seriousness and medical emphasis of podiatry work and the level of skills and knowledge required [16].

Our study complements research exploring occupational therapy career choice motivation for mature students where financial pressure was identified as the key factor deterring students choosing this career [17]. The loss of the NHS bursary was seen as the likely reason that accelerated the decline in undergraduate applications to podiatry courses [10, 16]. Podiatry was greatly affected because of the high proportion of mature students [4, 16, 18] who are more ‘debt-adverse’ than younger students and are more likely to have responsibilities which require funding [19]. Therefore, our finding relating to the importance of student bursaries among the Post-FC group is unsurprising. Minority ethnic students particularly from lower income groups are more averse to taking out loans [18] and therefore the bursary removal was likely to affect these students more. The Saks report confirmed that there had been an increase in student recruitment to podiatry courses in the current academic year and highlighted the ‘welcome reinstatement of the bursaries’ [4]. From September 2020, students starting or continuing an undergraduate or postgraduate podiatry course could apply for the NHS Learning Support Fund allowance. Our research took place in early 2021, and as such, public awareness of the fund may still be growing. That there has been an increase in student recruitment [4] suggests that awareness of the fund has improved since we undertook data collection, in addition to the vast HEE and Office for Students funded careers activity in recent years. Nevertheless, it is critical that public awareness of this fund is widespread so that different population groups (e.g., mature students; minority ethnic groups) do not see financial challenges as a barrier to choosing podiatry as their career.

There is a synonymous link between mature students and Post-FC participants, as in our study, 94% of our Post-FC group were older than 25 years of age. Interestingly, although the Office for Students stated that older podiatry students have a greater interest in private practice and potentially earning a large salary [16], such findings were not evident in our study (Table 4). For example, 86.1% of our Post-FC respondents agreed/strongly agreed that working in the NHS was a key reason for choosing podiatry. With there being a minimum of a 19% NHS vacancy rate predicted in England for podiatry by 2025 [20] and with 60% of members of the Royal College of Podiatry working in the private sector [4], it may be prudent for NHS recruitment campaigns to either: i) increase recruitment of Post-FC podiatrists as they may want to work in the NHS, or ii) develop strategies to encourage Pre-FC podiatrists to be motivated to work in the public sector.

### Influences

Post-FC participants recognised *own patient experiences* (their own experience or that of a relative receiving care from the profession) as a more important personal influence than the Pre-FC group. In the study by Byrne [21], mature students reported a proportionally higher amount of exposure to occupational therapy through personal life experiences than the rest of the cohort. More generally, in accordance with recent research [10, 22], a high proportion of our study sample (46 and 50.1% for Pre-FC and Post-FC, respectively) were influenced by their own personal (or relatives) podiatry treatment. These findings highlight the opportunity for qualified podiatrists; they can take on the role of career ambassador when meeting patients. This message needs to be conveyed to all podiatrists through the NHS, private practice, HEE and the Royal College of Podiatry. Activating this extensive workforce to be the ambassadors for the profession so that they see every patient and relative as a future podiatrist and to overtly prioritise work experience and university clinics opening doors for work experience.

Unsurprisingly, and similar to Craik et al. [22], we found school or college sources of influence to be approaching statistical differences: these sources were more important for the Pre-FC group. However, career advisors' lack of awareness of the profession was reported to be a substantial barrier for the Pre-FC group. In accordance with past research, careers advisors, and to a lesser extent, teachers, were reported to not have a strong influence on Pre-FC’s choice to engage in podiatry [23, 24, 25]. However, previous employment in healthcare was perceived to be an important influence for the Post-FC group to engage with the profession. This was a similar finding to Craik et al. [22], who suggested that the higher number of mature students in occupational therapy may be partly owing to students only hearing about the profession through their work in health care settings and not when they first make their career choice at school or college. Career advisors are a vital conduit to the successful recruitment of students to AHPs [21], and therefore, they need to have a good level of knowledge and understanding about the podiatry profession. With medicine and nursing still primarily promoted as the key healthcare careers in schools [9], career advisors can use the familiarity of these careers to introduce students to AHPs, including podiatry.

For Pre-FC participants, *someone in the profession I saw/met who was a really good role model for me* and *my own research* were considered more important than school or college sources. The importance of seeing AHP role models and the impact on career choice, has been explored in the literature, especially *the lack of* role models for minority ethnic individuals in particular professions, such as physiotherapy [26, 27]. Despite increases in the ethnic diversity of podiatry students in recent years [6], the Saks report [4] recommended improving efforts to recruit a more diverse (including ethnicity and gender) podiatry student population. In our study, the majority of the sample were white (85 and 62% for Post-FC and Pre-FC participants, respectively) and the importance of seeing role models in the profession suggests that more ethnic minority role models in podiatry are needed to support student recruitment.

### Barriers

*Job availability* was perceived to be an important barrier for the Post-FC group and *careers advisors' lack of awareness of the profession* and *misconception of profession* were reported to be more important barriers for the Pre-FC group. Previous research has shown that podiatry is an attractive career path to mature students as it leads to ‘almost certain employment’ following completion of undergraduate podiatry courses [4, 9, 10]. The importance of employment for Post-FC participants was shown in our study as the more mature students reported this to be of greater importance than Pre-FC participants. Job availability is not considered a pertinent reason for selecting different AHP careers [28, 29]. However, although it may not be a key motivation, ensuring that job availability is viewed positively especially owing to the current podiatry recruitment challenges, is important especially for the Post-FC group.

Misconceptions around the profession, highlighted also in answers to the open questions, suggests that marketing needs to emphasise the ‘extensive, diverse and interesting’ scope of practice [4]. The Saks report [4] also suggested that podiatry is not portrayed sufficiently as an appealing or important career. Our study was unable to determine why individuals working as an AHP overlooked the opportunity to engage in podiatry. Despite noting that there is an opportunity to maximise on the territory of the foot [30] once invested in the profession [31], the perceived status of the profession may lead to this never being actualised. Through addressing the perceived status of the podiatry profession by contemporary research, it may be possible to target marketing to attract applicants. The Saks report [4] highlighted the image of podiatry as a profession being perceived negatively by the public. Tollafield [32] suggested overcoming the ‘ugh factor’ associated with working with feet with an emphasis on function not condition; podiatrists help return people to activity and occupation as a slogan, not podiatrists’ work with ingrowing toenails and ulcers. Current research is being undertaken to understand perceptions of the human foot among social media users and this may help understand the ‘ugh factor’. But more broadly, research is needed to understand the relationship of profession status, ‘ugh factor’ and podiatry as a career choice. It is clear that other AHPs whilst larger in numbers, also have greater visibility and a better known profile, for example physiotherapists and paramedics. Media marketing strategies for podiatry should be implemented that not only increases the public awareness of the profession, but also enhances the government and other health professionals’ understanding of podiatry [4]. In addition, that there has been an increased interest in health and care professions during the Covid-19 pandemic and changing employment circumstances and priorities [33] suggests now is the right time to market podiatry to individuals looking for a career change.

#### Strengths and limitations

It is important to contextualise our findings in light of the strengths and limitations to the study. The study was conducted prior to the publication of the Podiatry Career Framework [34] and Standards for the Foot Health Workforce [35]. These frameworks may influence the decision to study podiatry among podiatry associate professions in future years. Although conducting the study in February and March 2021 provided a fascinating insight into views of podiatry students during the COVID-19 pandemic, participant responses will have been influenced by the unique academic and professional conditions which participants experienced [36].

Despite the questionnaire being piloted, it had not been validated. Furthermore, recall ability is a limitation of questionnaires [37], and this is likely to have affected the participants in our study owing to new impressions formed on the course influencing perceptions of podiatry. The study sample lacked ethnic diversity as 75% of respondents were white. However, our sample was fairly representative of qualified podiatrists in the UK registered with the HCPC [38], with minority ethnic males, for example, comprising 3.5% of the study sample. Our sample was a relatively small self-selecting proportion of all podiatry students in England. Therefore, the findings cannot be seen to represent the views of the wider podiatry student population [39]. There are a number of existing studies exploring career choice motivations in other AHPs but they focus on one university or geographical area (for example, Craik et al. [23]). Finally, due to the substantial number of analyses, and the stringency in reporting the results to minimise the risk of type I error via the bonferroni technique, the study demonstrated a lack of statistical findings. A more refined questionnaire (fewer items) and a larger sample size may enable future research studies to elicit statistically significant findings. A real strength of our study was that it was a first national questionnaire about podiatry students, the results of which provide a data set for future studies exploring podiatry as a career choice.

## Conclusion

The Saks report [4] mentioned the need for research exploring why students chose podiatry as their career route. Our study has afforded a nationwide insight into the motivations, sources of influence and barriers among people who chose podiatry as their career. This is the first study with national reach in this field and reveals areas for future focus in marketing for pre-registration course recruitment. This study has highlighted that individuals are choosing podiatry at all life stages and ages and yet there were a number of similarities between the two groups (pre and post a first career) in terms of motivations and sources of influence. This suggests that marketing and marketing channels can be applicable to all individuals and presents a broad opportunity for recruiting individuals into podiatry to meet the workforce need. Further studies are needed to explore more deeply the reasons podiatry is overlooked as a career and how to maximise the opportunities in recruitment. Finally, the influence of personal encounters with podiatrists shows the transformational role podiatrists can have in recruiting to the profession. The research suggests that an essential first step is to activate the profession into an ambassadorial mindset to actively participate in recruiting the next generation of podiatrists to reignite recruitment to the profession at a time when it has never been more needed or important with an ageing demographic and increased diabetes and vascular need in the modern world.

## Supplementary Information

**Additional file 1.** Questionnaire - An example of the full questionnaire which was distributed to students is provided.

**Additional file 2: Supplementary Table 1.** Day-today context for engaging in podiatry for both Pre-FC and Post-FC participants and **Supplementary Table 2.** How information on podiatry is accessed for both Pre-FC and Post-FC participants and **Supplementary Table 3.** Professional, personal and understanding barriers for engaging in podiatry for both Pre-FC and Post-FC participants.

## Data Availability

The datasets used and/or analysed during the current study are available from the corresponding author on reasonable request.

## Abbreviations

AHP: Allied health professionals
HCPC: Health and Care Professions Council
HEE: Health Education England
NHS: National Health Service
Pre-FC: Questionnaire respondents who engaged in podiatry *before* their first career in employment
Post-FC: Questionnaire respondents who engaged in podiatry *after* their first career in employment
