# Supplementary material for: Motivations, sources of influence and barriers to being a podiatrist: a national questionnaire of student views
Source: J Foot Ankle Res. 2022 May 28;15:41. doi: 10.1186/s13047-022-00551-6 (PMC9142343; doi:10.1186/s13047-022-00551-6)
Supplement: Supplementary file 1 — Additional file 1. Questionnaire - An example of the full questionnaire which was distributed to students is provided. [file 13047_2022_551_MOESM1_ESM.docx]

Questionnaire

**1 I have read the participant information page and I am happy to participate.**

**I understand my participation is voluntary and I may withdraw at any time without my legal rights being affected.**

**I understand that by completing and submitting this questionnaire I am consenting to be part of the research study and for my data to be used as described.**

**2 What is your gender?**

**3 Are you studying full-time or part-time?**

**4 Which year of study are you in?**

**5 What is your age band?**

**6 Which option best describes your ethnic group?**

**7 Are your day-to-day activities limited because of a physical or mental health problem or disability which has lasted, or is expected to last at least 12 months? Include limitations you would experience without medication or treatment and limitations that are due to old age.**

**7a If you have responded 'Yes' above. Do you have any of the following impairments or health conditions?**

- a physical impairment such as difficulty using your arms or mobility difficulties which require you to use a wheelchair or other mobility aid
- a sensory impairment such as serious vision difficulties or blindness, or deafness
- a mental health condition, such as depression or schizophrenia that has lasted or is expected to last 12 months or more
- a learning difficulty or disability such as down’s syndrome or dyslexia or
- a cognitive impairment such as autistic spectrum disorder diagnosed as having HIV, Cancer or Multiple Sclerosis
- Other long-term illness or health condition that has lasted or is expected to last 12 months or more

**8 My chosen professional area is:**

**9 Which other health professions did you consider? (Please tick all which apply).**

**10 At what stage did you make the decision to become this profession?**

**11 When accessing information about your profession which website(s) did you use? (Please tick all which apply).**

**12 Below are 10 statements about how your perceptions about your personal interests and affinities could influence your AHP career choice. For each statement mark the answer that best describes how you feel. My career choice was influenced by choosing a profession...**

That reflects my academic interests and abilities

Based on my interest in a particular area of the profession (e.g. art, language or technology)

That is challenging/demanding

That is intellectually stimulating

With high-profile responsibilities

That suits my personal qualities and values

That has a good public image and prestige

Which is respected in my culture

Which is suited to my religious background

Which has supportive attitudes to an individual’s culture and background

Any comments you wish to add relating to your answers

**13 Below are 15 statements about how your perceptions about the professional aspect of your profession could influence your AHP career choice. For each statement mark the answer that best describes how you feel. My career choice was influenced by…**

Choosing a vocational degree and a profession

Choosing a course that offers student bursaries and finance

Regular working hours of the profession allowing for a good work/life balance

The salary

The potential for job security

The opportunity to be entrepreneurial

Good job availability and employment opportunities

The opportunity to work in the private sector

The opportunity to work in the public sector

The opportunity to work in the NHS

The opportunity to work in healthcare

The ability to move anywhere or to work overseas

The good opportunities for my career advancement

The further educational/research/teaching opportunities

The opportunity to practise in different work environments

Any comments you wish to add relating to your answers.

**14 Below are 10 statements about how your perceptions about the day-to-day content of the job could influence your AHP career choice. For each statement mark the answer that best describes how you feel. My career choice was influenced by choosing a profession...**

Where I can help others and make a contribution to society

Where I can use my skills to improve the quality of life for a patient/service user

Where I can engage in/build human relationships

Where I can work with a range of patients/service users

Where I can help a specific group of people

That is fulfilling and satisfying

That is exciting and involves working under pressure

Which is a non-sedentary profession

Where I can work in a team

That offers variety and is more than just routine

Any comments you wish to add relating to your answers.

**15 Are there any other motivations?**

**16 Below are 14 personal and educational sources of influence impacting on AHP career choice. For each statement mark the answer that best describes how you feel. (Please select N/A if the opportunity did not arise - for example if you did not hear about the profession from a teacher). My career choice was influenced by hearing about the profession through…**

My previous job in healthcare

Someone in the profession I saw/met who was a really good role model for me

A family member or a relative

A friend

Someone who works closely with the profession

My own experience of being a patient/a relative receiving care from the profession

My own research

A teacher

An individual in the profession visiting the school/college

A future careers programme run by my school/college

A careers advice person

Voluntary work in healthcare settings

Work shadowing experiences with the profession

My first university degree

Any comments you wish to add relating to your answers.

**17 Below are 11 media and marketing sources of influence impacting on AHP career choice. For each statement mark the answer that best describes how you feel. (Please select N/A if the opportunity did not arise - for example if you did not hear about the profession from a television programme). My career choice was influenced by hearing about the profession through…**

Seeing people like me doing the profession represented in the media

Social media

The WOW show

Television programmes including sports matches or the news

Print adverts

Television adverts

National bodies

Information I got from universities

Attending a university open day

A careers fair

National days (e.g. National ODP day) at a hospital, university or school/college

Any comments you wish to add relating to your answers.

**18 Are there any other sources of influence?**

**19 What do you feel is the public understanding of your profession (positive or negative aspects)?**

**20 Below are 8 statements about potential personal barriers to entering an AHP career. For each statement mark the answer that best describes your experience with the barrier. A potential barrier in my career decision making was presented by...**

A lack of prestige associated with the profession

Perceived lack of representation in the profession in terms of gender

Perceived lack of representation in the profession in terms of ethnicity

Perceived image of who does the profession

Perceived difficulty of getting onto the course

Perception of the course as difficult (including too academic)

A concern around accessibility of the course/profession in terms of physical or mental disability

The emotional burden of the role

Outside obligations I have

Any comments you wish to add relating to your answers.

**21 Below are 8 statements about potential professional barriers to entering an AHP career. For each statement mark the answer that best describes your experience with the barrier. A potential barrier in my career decision making was presented by...**

Geographical location of training course

Not being able to access funding for a second degree

Funding the course and availability of financial support

Cost of training whilst undertaking the course (e.g. costs of placements)

High workload and pressure of training

Perceived working conditions within the NHS

Attitudes present in the NHS workplace

Job availability

Any comments you wish to add relating to your answers.

**22 Below are 6 statements about potential understanding of the role barriers to entering an AHP career. For each statement mark the answer that best describes your experience with the barrier. A potential barrier in my career decision making was presented by...**

Poor access I had to work shadowing experience

Limited awareness of the existence of the profession

Challenges in accessing information about the profession

Limited amount of information about the profession available

Lack of understanding about the profession from careers advisors at school/college

Misconceptions around the profession and what the role involves

Any comments you wish to add relating to your answers.

**23 Are there any other potential barriers?**

**24 From what you know now as a student on your programme, what advice would you give someone interested in this profession?**

**25 Do you feel you made the right choice of profession?**
