# Supplementary material for: Motivations, sources of influence and barriers to being a podiatrist: a national questionnaire of student views
Source: J Foot Ankle Res. 2022 May 28;15:41. doi: 10.1186/s13047-022-00551-6 (PMC9142343; doi:10.1186/s13047-022-00551-6)
Supplement: Supplementary file 2 — Additional file 2: Supplementary Table 1. Day-today context for engaging in podiatry for both Pre-FC and Post-FC participants and Supplementary Table 2. How information on podiatry is accessed for both Pre-FC and Post-FC participants and Supplementary Table 3. Professional, personal and understanding barriers for engaging in podiatry for both Pre-FC and Post-FC participants. [file 13047_2022_551_MOESM2_ESM.docx]

Supplementary Tables 1, 2 and 3

**Supplementary Table 1:** Day-to-day context for engaging in podiatry for both Pre-FC and Post-FC participants

| **Motivation** | **Group** | **Median (IQR)** | **Mean**  **rank** | **U** | **z** | **p** | **Effect size (r)** |
| --- | --- | --- | --- | --- | --- | --- | --- |
| **Contribution to society** | **Pre-FC** | 5 (4-5) | 55.09 | 1479.5 | -1.059 | 0.290 | 0.10 |
|  | **Post-FC** |  | 60.24 |  |  |  |  |
| **Skills to improve quality of life** | **Pre-FC** | 5 (5-5) | 55.03 | 1476.5 | -1.226 | 0.220 | 0.11 |
|  | **Post-FC** |  | 60.28 |  |  |  |  |
| **Build human relationships** | **Pre-FC** | 5 (4-5) | 58.03 | 1623.5 | -0.010 | 0.992 | 0.00 |
|  | **Post-FC** |  | 57.98 |  |  |  |  |
| **Work with a range of service users** | **Pre-FC** | 5 (4-5) | 57.76 | 1613 | -0.082 | 0.934 | 0.01 |
|  | **Post-FC** |  | 58.18 |  |  |  |  |
| **Work with specific groups** | **Pre-FC** | 4 (4-5) | 58.85 | 1582.5 | -0.256 | 0.798 | 0.02 |
|  | **Post-FC** |  | 57.35 |  |  |  |  |
| **Fulfilling** | **Pre-FC** | 5 (4-5) | 54.22 | 1436 | -1.393 | 0.164 | 0.13 |
|  | **Post-FC** |  | 60.91 |  |  |  |  |
| **Working under pressure** | **Pre-FC** | 4 (4-5) | 59.78 | 1536 | -0.544 | 0.587 | 0.05 |
|  | **Post-FC** |  | 56.63 |  |  |  |  |
| **Non-sedentary profession** | **Pre-FC** | 4 (3-5) | 56.98 | 1574 | -0.303 | 0.762 | 0.03 |
|  | **Post-FC** |  | 58.78 |  |  |  |  |
| **Work in a team** | **Pre-FC** | 4 (4-5) | 58.31 | 1609.5 | -0.096 | 0.924 | 0.01 |
|  | **Post-FC** |  | 57.76 |  |  |  |  |
| **Offers variety** | **Pre-FC** | 5 (4-5) | 56.36 | 1543 | -0.542 | 0.588 | 0.05 |
|  | **Post-FC** |  | 59.26 |  |  |  |  |

*Note:* IQR, interquartile range (25^th^ – 75^th^ percentiles); Pre-FC, before first career; Post-FC, after first career

**Supplementary Table 2:** How information on podiatry is accessed for both Pre-FC and Post-FC participants

| Resource | **Group** | **Median**  **(IQR)** | **Mean**  **rank** | **U** | **z** | **p** | **Effect size (r)** |
| --- | --- | --- | --- | --- | --- | --- | --- |
| **Health Website** | **Pre-FC** | 1 (0-1) | 60.54 | 1448 | -1.063 | 0.288 | 0.10 |
|  | **Post-FC** |  | 55.13 |  |  |  |  |
| **Job Website** | **Pre-FC** | 0 (0-0) | 56.54 | 1552 | -0.372 | 0.710 | 0.03 |
|  | **Post-FC** |  | 58.25 |  |  |  |  |
| **University Website** | **Pre-FC** | 1 (1-1) | 59.10 | 1520 | -0.629 | 0.530 | 0.06 |
|  | **Post-FC** |  | 56.25 |  |  |  |  |
| **Social media** | **Pre-FC** | 0 (0-0) | 58.32 | 1559 | -0.314 | 0.754 | 0.03 |
|  | **Post-FC** |  | 56.86 |  |  |  |  |
| **Other** | **Pre-FC** | 0 (0-0) | 56.06 | 1528 | -0.773 | 0.439 | 0.07 |
|  | **Post-FC** |  | 58.63 |  |  |  |  |

*Note:* IQR, interquartile range (25^th^ – 75^th^ percentiles); Pre-FC, before first career; Post-FC, after first career

**Supplementary Table 3:** Professional, personal and understanding barriers for engaging in podiatry for both Pre-FC and Post-FC participants

|  | **Barrier** | **Group** | **Median**  **(IQR)** | **Mean rank** | **U** | **z** | **p** | **Effect size (r)** |
| --- | --- | --- | --- | --- | --- | --- | --- | --- |
| **Professional barriers** | **Geographical location** | **Pre-FC** | 3 (2-4) | 50.13 | 1225.00 | -1.148 | 0.251 | 0.11 |
|  |  | **Post-FC** |  | 56.92 |  |  |  |  |
|  | **Lack of funding for second degree** | **Pre-FC** | 4 (2-5) | 34.84 | 569.50 | -0.706 | 0.480 | 0.07 |
|  |  | **Post-FC** |  | 38.34 |  |  |  |  |
|  | **Financial support** | **Pre-FC** | 4 (2-5) | 47.78 | 1108.50 | -1.747 | 0.081***** | 0.16 |
|  |  | **Post-FC** |  | 58.18 |  |  |  |  |
|  | **Cost of training** | **Pre-FC** | 4 (2-5) | 48.41 | 1124.00 | -1.267 | 0.205 | 0.12 |
|  |  | **Post-FC** |  | 55.94 |  |  |  |  |
|  | **High workload** | **Pre-FC** | 4 (2-4) | 54.4 | 1440.50 | -0.904 | 0.366 | 0.08 |
|  |  | **Post-FC** |  | 59.84 |  |  |  |  |
|  | **Perceived working conditions** | **Pre-FC** | 3 (2-4) | 53.22 | 1367.00 | -0.520 | 0.603 | 0.05 |
|  |  | **Post-FC** |  | 56.3 |  |  |  |  |
|  | **Attitudes to NHS workplace** | **Pre-FC** | 3 (2-4) | 52.1 | 1315.5 | -0.569 | 0.570 | 0.05 |
|  |  | **Post-FC** |  | 55.43 |  |  |  |  |
|  | **Job availability** | **Pre-FC** | 3 (2-4) | 49.34 | 1191 | -2.037 | 0.042***** | 0.19 |
|  |  | **Post-FC** |  | 61.68 |  |  |  |  |
| **Personal Barriers** | **Lack prestige** | **Pre-FC** | 3 (2-4) | 58.51 | 1149.50 | -1.511 | 0.131 | 0.14 |
|  |  | **Post-FC** |  | 49.66 |  |  |  |  |
|  | **Lack of representation (gender)** | **Pre-FC** | 2 (2-3) | 52.86 | 1124 | -0.762 | 0.446 | 0.07 |
|  |  | **Post-FC** |  | 48.72 |  |  |  |  |
|  | **Lack of representation (ethnicity)** | **Pre-FC** | 2 (2-3) | 52.27 | 1143.5 | -0.554 | 0.579 | 0.05 |
|  |  | **Post-FC** |  | 49.22 |  |  |  |  |
|  | **Perceived image** | **Pre-FC** | 2 (2-4) | 53.76 | 1271 | -0.384 | 0.701 | 0.04 |
|  |  | **Post-FC** |  | 51.54 |  |  |  |  |
|  | **Perceived difficulty of profession** | **Pre-FC** | 3 (2-4) | 53.76 | 1409 | -0.812 | 0.417 | 0.08 |
|  |  | **Post-FC** |  | 58.63 |  |  |  |  |
|  | **Perceived course difficulty** | **Pre-FC** | 3 (2-4) | 57.09 | 1470.5 | -0.332 | 0.740 | 0.04 |
|  |  | **Post-FC** |  | 55.11 |  |  |  |  |
|  | **Lack of accessibility** | **Pre-FC** | 3 (2-4) | 47.24 | 1085.5 | -0.087 | 0.931 | 0.01 |
|  |  | **Post-FC** |  | 47.72 |  |  |  |  |
|  | **Emotional burden** | **Pre-FC** | 3 (2-4) | 54.13 | 1409 | -0.254 | 0.800 | 0.02 |
|  |  | **Post-FC** |  | 55.63 |  |  |  |  |
|  | **Outside obligations** | **Pre-FC** | 3 (2-5) | 47.85 | 1120 | -1.698 | 0.090 | 0.16 |
|  |  | **Post-FC** |  | 57.83 |  |  |  |  |
| **Understanding barriers** | **Shadowing after first career** | **Pre-FC** | 3 (2-5) | 52.03 | 1078.5 | -0.838 | 0.402 | 0.08 |
|  |  | **Post-FC** |  | 47.35 |  |  |  |  |
|  | **Awareness of profession** | **Pre-FC** | 4 (2-4) | 59.08 | 1364 | -0.917 | 0.359 | 0.09 |
|  |  | **Post-FC** |  | 53.65 |  |  |  |  |
|  | **Accessing information on profession** | **Pre-FC** | 3 (2-4) | 59.86 | 1133.5 | -1.766 | 0.077 | 0.16 |
|  |  | **Post-FC** |  | 49.58 |  |  |  |  |
|  | **Limited information on profession** | **Pre-FC** | 3 (2-4) | 57.66 | 1285 | -0.955 | 0.340 | 0.09 |
|  |  | **Post-FC** |  | 52.07 |  |  |  |  |
|  | **Career advisors lack of awareness** | **Pre-FC** | 4 (3-5) | 51.23 | 599.5 | -2.889 | **0.004** | 0.27 |
|  |  | **Post-FC** |  | 36.13 |  |  |  |  |
|  | **Misconception of profession** | **Pre-FC** | 4 (2-5) | 62.24 | 1164 | -2.06 | 0.039* | 0.19 |
|  |  | **Post-FC** |  | 50.08 |  |  |  |  |

*Note:* IQR, interquartile range (25^th^ – 75^th^ percentiles); Pre-FC, before first career; Post-FC, after first career

Bold p values denotes significant difference between groups following Bonferroni adjustment (p = 0.008)

*Denotes p values that are approaching a significant difference between groups following a Bonferroni adjustment (p = .006)
